# Executive Functions and Language: Their Differential Influence on Mono- vs. Multilingual Spelling in Primary School

**DOI:** 10.3389/fpsyg.2019.00097

**Published:** 2019-02-06

**Authors:** Sophia Czapka, Annegret Klassert, Julia Festman

**Affiliations:** ^1^Research Group: Diversity and Inclusion, Human Sciences Faculty, University of Potsdam, Potsdam, Germany; ^2^Leibniz-Center General Linguistics, Berlin, Germany; ^3^University of Applied Sciences Clara Hoffbauer, Potsdam, Germany; ^4^Department of Primary and Secondary Education, Pedagogical University Tyrol, Innsbruck, Austria

**Keywords:** bilingualism, spelling, literacy acquisition, executive functions, lexicon size, primary school

## Abstract

We aimed at unveiling the role of executive functions (EFs) and language-related skills in spelling for mono- versus multilingual primary school children. We focused on EF and language-related skills, in particular lexicon size and phonological awareness (PA), because these factors were found to predict spelling in studies predominantly conducted with monolinguals, and because multilingualism can modulate these factors. There is evidence for (a) a *bilingual advantage* in EF due to constant high cognitive demands through language control, (b) a smaller mental lexicon in German and (c) possibly better PA. Multilinguals in Germany show on average poorer German language proficiency, what can influence performance on language-based tasks negatively. Thus, we included two spelling tasks to tease apart spelling based on lexical knowledge (i.e., word spelling) from spelling based on non-lexical strategies (i.e., non-word spelling). Our sample consisted of heterogeneous third graders from Germany: 69 monolinguals (age: *M* = 108 months) and 57 multilinguals (age: *M* = 111 months). On less language-dependent tasks (e.g., non-word spelling, PA, intelligence, short-term memory (STM) and three EF tasks testing switching, inhibition, and working memory) performance of both groups did not differ significantly. However, multilinguals performed significantly more poorly on tasks measuring German lexicon size and word spelling than monolinguals. Regression analyses revealed that for multilinguals, inhibition was related to spelling, whereas switching was the only EF component to influence word spelling in monolinguals and non-word spelling performance in both groups. By adding lexicon size and other language-related factors to the regression models, the influence of switching was reduced to insignificant effects, but inhibition remained significant for multilinguals. Language-related skills best predicted spelling and both language groups shared those variables: PA for word spelling, and STM for non-word spelling. Additionally, multilinguals’ word spelling performance was also predicted by their German lexicon size, and non-word spelling performance by PA. This study offers an in-depth look at spelling acquisition at a certain point of literacy development. Mono- and multilinguals have the predominant factors for spelling in common, but probably due to superior language knowledge, monolinguals were already able to make use of EF during spelling. For multilinguals, German lexicon size was more important for spelling than EF. For multilinguals’ spelling these functions might come into play only at a later stage.

## Introduction

Spelling *per se* is a highly crucial skill, because “[…] struggling with spelling production may result in students being demotivated, running out of time, having less time for planning or writing a shorter text” (Rønneberg and Torrance, 2017, p. 2). Hence, spelling influences the quality of a text, and often has an impact on the reader’s judgment of the writer’s competences. Reading texts full of spelling mistakes makes the comprehension of the content taxing and tiring, as it interrupts the perception of content. Thus, spelling is important for demonstrating subject-related competence, especially in the school context. Additionally, it has significant consequences, since the ability to spell correctly is also known to be a crucial criterion whether teachers recommend pupils for junior high school in the very selective German school system (Roos and Schöler, 2009).

Known predictors for literacy in general are specific language [such as phonological awareness (PA) and size of the mental lexicon] and cognitive skills [in particular executive functions (EFs)], but their role in spelling is not clear-cut. It also remains unknown if these predictors play the same role for multilinguals. Therefore, it is important to investigate not only the underlying internal factors influencing monolingual children’s spelling performance, as multilingual children make up a considerable part of the student body in Germany (e.g., 38% of primary school children in Berlin; Leerhoff et al., 2013) and are considered at risk for school failure (Zöller et al., 2006; Ditton and Krüsken, 2009; OECD, 2014). A main contributor to this risk is the on average lower proficiency in German, the language of schooling (Niklas et al., 2011). This impacts negatively on their reading performance. From primary school throughout the children’s school career it is on average lower compared to their monolingual peers’ (Müller and Stanat, 2006; Marx and Stanat, 2012). For spelling, findings showed multilinguals’ similar (Roos and Schöler, 2009; Chudaske, 2012) or poorer performance (Schründer-Lenzen and Merkens, 2006) compared to their monolingual peers.

In sum, the influence of language-related and cognitive predictors of spelling, in particular for multilingual children, are largely unknown and findings for multilinguals’ spelling competences are mixed. As research on the acquisition of spelling is considered to be rather anglo-centric (Wimmer and Landerl, 1997) and the English orthography is less consistent than German, it is called for an investigation of these different language-related and cognitive predictors for spelling in German.

### Spelling

Contemporary accounts of writing include both linguistic and cognitive processes which are functionally integrated when writing (Abbott and Berninger, 1993; Berninger and Amtmann, 2003). These language skills comprise for example PA (i.e., the ability to perceive the phonological structure of words and manipulate elements of spoken language), as well as alphabetical, morphological, and lexical knowledge (Lubin et al., 2016). Being able to produce the correct, orthographic spelling of words requires a number of linguistic processes to be executed (e.g., phonological and morphological analysis of the word, translation into graphemes; Alamargot and Chanquoy, 2001; Treiman, 2017) and orchestrated by cognitive processes prior to graphomotor execution (i.e., the writing process itself).

Beginning writers’ spelling in alphabetic orthographies like German is phonemic (see Ziegler and Goswami, 2006) and thus relies on phoneme–grapheme conversion. This is one of two different procedures to spell as suggested by the *dual-route account of orthographic retrieval* (e.g., Barry, 1994). Spelling via this *non-lexical route* relies on the direct translation of phonemes to their corresponding graphemes–so called phoneme–grapheme conversion (e.g., ‘enough’ [I’nΛf] could result in the phonemic spelling ‘inaf’). With increasing training and through experience with printed language (Ehri, 2005, 2014), orthographic learning takes place, meaning the phonology and orthography of a word become connected, and an orthographic representation is stored in memory. These orthographic word forms build a separate orthographic lexicon (that is independent of the phonological forms stored in memory; Berninger et al., 2006), what allows direct retrieval of word forms from that lexicon. This route is the *lexical route* of spelling (the second procedure of the *dual-route account*). The direct access makes spelling more efficient (meaning correct and fast) (Berninger et al., 2006). However, this route can only process familiar words that already have lexical representations. Non-words and unfamiliar words are spelled via the non-lexical route, which is costlier than accessing whole word forms, because phoneme–grapheme conversion and orthographic rules need to be aligned. In conclusion, an extensive orthographic lexicon is essential for efficient word spelling (Roos and Schöler, 2009).

The *componential model of writing* (Schoonen et al., 2002; Harrison et al., 2016) broadens the complexity of the writing skill and includes also more cognitive components. It distinguishes between lower-level writing skills, comprising handwriting, punctuation and spelling, and higher-level writing skills, i.e., planning, formulating/composition, revising (McCutchen, 1996). Lower-level writing skills are acquired first in writing acquisition and initially lack an automatized execution. Hence, they require conscious control through EF (see section “EF and Spelling”) and use up cognitive resources (Bourdin and Fayol, 1994; Grabowski, 2010). Cognitive resources comprise many mental processes (e.g., attention, memory, motor control and EF; Franconeri et al., 2013) and are limited in their capacity (following the *capacity theory of writing* by McCutchen, 1996, 2011). Hence, children who are still struggling with graphomotor execution have problems in spelling an entire word correctly, since the handwriting process itself takes up too many cognitive resources (Pontart et al., 2013). Similarly, if spelling draws on children’s attention, it draws processing resources away from higher-level processes and few resources are left for high-quality text composition. Therefore, children need to train spelling to reach a more automatic execution of these lower-level writing skills (for an overview, see Gerth et al., 2016). When writers become more advanced (around the age of 14), the automatization of these lower-level processes frees cognitive resources for higher-level processes such as sentence- and text-level processes (McCutchen, 1996). Even for advanced writers, writing draws on cognitive resources, because different lower-level and higher-level processes need to be coordinated concurrently (Alamargot and Chanquoy, 2001).

In sum, spelling is a developing skill especially in primary school children, as spelling is taught mainly in the school context. Spelling draws on a number of different cognitive and language-related processes. In the following sections, we will first describe the links between the three EF components proposed by Miyake and Friedman (2012) (i.e., switching, inhibition and updating) and spelling, before we highlight the impact of bi-/multilingualism on EF. Finally, we will portray the links between the language-related skills and spelling in more detail, with a specific focus on the role of lexicon size and PA for multilinguals.

### EF and Spelling

Executive functions are a family of cognitive control mechanisms that regulate thought and action. These effortful, mental processes can be divided into three core functions: first, switching (or shifting) describes the ability to switch flexibly between mental sets or tasks. Secondly, inhibition is the ability to suppress dominant or irrelevant information or behavior to maintain a task goal. Thirdly, working memory (WM or updating) is the ability to manipulate mentally stored information (Miyake and Friedman, 2012; Diamond, 2013).

Executive functions are an important prerequisite for school success: they have been shown to predict school readiness and school performance (St Clair-Thompson and Gathercole, 2006; Diamond, 2013; Zorza et al., 2017), reading and mathematics (Best et al., 2011) and also to affect writing (e.g., Kellogg, 1996; Monette et al., 2011). Despite the clearly crucial contribution of EF to literacy development in general, there is still no consistent picture in regard to the impact of EF on spelling as one important literacy subprocess. To the best of our knowledge, there are only very few studies that investigated the direct influence of concrete EF components (switching, inhibition and WM) on spelling. In the following sections, we will give a short overview of their findings and—due to the very low number of specific studies on spelling—we occasionally extend the review to literacy more generally.

#### Influence of Switching on Spelling

During spelling different processes need to be coordinated (as described above) and the writer needs to switch effectively between them to write fluently and correctly. For example, one needs to translate phonemes into graphemes (non-lexical route) or retrieve the spelling of the word from orthographic memory (lexical route), apply orthographic rules, compare phonemic and lexical word forms, and finally plan and execute graphomotor processes (Lubin et al., 2016). Third grade students are at a developmental stage of transition from phonemic spelling to extending their orthographic lexicon allowing the lexical route to be used more frequently (von Suchodoletz et al., 2017). This requires children to switch between these coexisting routes (non-lexical and lexical route) and even include, e.g., morphological strategies to support their spelling activities. Good switching abilities allow for flexible application of these different options to changing task demands during spelling.

Two studies confirmed a direct connection between switching and spelling. Lubin et al. (2016) investigated how switching, inhibition and WM influence spelling in primary school children. Their study was based on a group of native French-speaking fourth graders in France. For the switching task, children had to switch between counting up- and downwards depending on a cue. They found that switching was the only EF component explaining variance in their French spelling task. Von Suchodoletz (2017) conducted a cross-sectional study on attention shifting, assessed with a card sorting task, and spelling in German first, third and eighth graders. They found that better switching abilities were related to superior general spelling skills, in particular in third graders.

In two other studies on literacy in English, switching was found to predict reading, written expression and spelling of English at grade one to three (Altemeier et al., 2008) and also writing in the subsequent 6 years (Berninger et al., 2016). Switching in these studies was measured with a task called *rapid automatized switching* that combines a rapid naming task with increased EF demands through switching categories (e.g., naming alternatingly numbers and letters). However, because *rapid automatized naming* (RAN) on its own predicts reading and possibly writing (see below), we believe that it is difficult to tease apart the influence of RAN and switching when using rapid automatized switching as predictor.

#### Influence of Inhibition on Spelling

In our study we refer to inhibition as the ability to control interference at the level of perception; it is an involuntary and automatic reaction to or away from a stimulus (Diamond, 2013). According to Diamond (2013), this definition contrasts with other forms of inhibition like selective attention, which is a voluntary and active focus of one’s attention to or away from a stimulus, or self-control, i.e., the control of one’s behavior and emotions to resist temptation.

Writing involves many competitive processes that require inhibition of an irrelevant or incorrect choice to select the appropriate alternative. In spelling, inhibition should be involved in choosing between lexical and non-lexical spelling routes, or in the selection of neighborhood word competitors which is suggested to be easier the more precise the orthographic knowledge is (Perfetti, 2017).

There is some evidence that inhibition is involved in reading-related skills (Blair and Razza, 2007) and literacy in general (assessed with general scores for reading and spelling; St Clair-Thompson and Gathercole, 2006; Allan et al., 2014). However, these general findings with respect to literacy do not allow us to draw conclusions on spelling. Concerning spelling more specifically, there seems to be hardly any evidence for an influence of inhibition on spelling: according to Altemeier et al. (2008), who tested English-speaking children longitudinally in the first 4 years of school, inhibition (assessed with a Color-Word interference test) additionally to rapid automatized switching (see section “Influence of Switching on Spelling”) predicted reading and writing, but not spelling. Also, Lubin et al. (2016) reported no impact of inhibition (measured with an Opposite World task) on spelling in French monolingual children. However, Lubin et al. (2016) discussed the possibility that in more speeded conditions or under conditions in which automatic processes need to be inhibited, inhibition abilities might become relevant.

#### Influence of WM on Spelling

WM is the third EF component identified by Miyake et al. (2000) and a complex structure itself. In Baddeley’s model of WM, the ability to manipulate mentally stored information (what we refer to as WM) is part of the so-called central executive (Repovš and Baddeley, 2006; Baddeley, 2010). Baddeley’s model additionally comprises separate functions for mental short-term storage: one for visual and spatial information, the visuo-spatial sketchpad, and one for verbal material, that is the phonological loop or phonological short-term memory (STM).

In writing research, WM is probably the most investigated EF component. The role of WM in proficient writing is described in Kellogg’s model (Kellogg, 1996), in which Baddeley’s central executive affects all sub-processes of writing (Alamargot and Chanquoy, 2001). WM plays a role in particular for higher-level writing skills, i.e., processes involved in text composition (e.g., planning, formulating, revising). WM is necessary for “active maintenance of multiple ideas, the retrieval of grammatical rules from long-term memory, and the recursive self-monitoring that is required during the act of writing” (Hooper et al., 2002). WM is also involved in spelling to dictation in the following way: while words and decoded sounds are stored temporarily in STM, processing of this information takes place in WM (orthographic information is retrieved from long-term memory, morphological rules are applied or phonemes translated to corresponding graphemes) and concurrently the writer needs to continue listening and updates the STM content, what is the task of WM (Strattman and Hodson, 2005).

Experimental support for the role of WM in spelling acquisition, however, is weak. Research findings suggest that WM and spelling are not connected in primary school children (see Lubin et al., 2016). Swanson and Berninger (1996) reported that in children at the age of 10 to 12, WM was rather connected to higher-level and STM to lower-level writing skills. Also, for older children, Vanderberg and Swanson (2007) found that in 15-year-olds, WM (the authors refer to the central executive) predicted all levels of writing (planning, translating, revision), except spelling. Berninger et al. (2010) used a global measure of WM that combined a non-word repetition task for STM and backward digit span for WM (testing the central executive). They found that WM influenced spelling from second to sixth grade, but this finding does not allow generalization for specific components of EF or WM, since STM and WM represent distinct cognitive functions. To summarize, the influence of WM on spelling seems to be crucial for higher-level tasks (Swanson and Berninger, 1996), which play only a very minor role for primary school children, since they are mainly acquiring lower-level writing skills.

#### Influence of Bi- and Multilingualism on EF

As we study predictors for mono- versus multilinguals’ performance, we need to take into account that multilingualism can affect EF positively (Adesope et al., 2010; Bialystok, 2015; Hilchey et al., 2015; Zhang, 2018). The reason for the so-called *bilingual advantage* is that a bilingual’s languages are constantly activated, what puts particular demands on cognitive control to activate the language currently in use and inhibit the non-target language (Green, 1998; Kroll et al., 2012; Spalek et al., 2014). The constant load on the EF system has been suggested to constitute a training and to lead to improved EF (Bialystok, 2015). Strongest evidence for a bilingual advantage has been found for inhibition tasks (Bialystok and Viswanathan, 2009; Poarch and van Hell, 2012; Poarch, 2018). For WM, there is evidence for a positive influence of bilingualism (Luo et al., 2013; Blom et al., 2014), but also against (Namazi and Thordardottir, 2010; Engel de Abreu, 2011). Finally, superior switching performance tested with card sorting tasks could be related to bilingualism (Bialystok and Martin, 2004; Wiseheart et al., 2016).

It is important to note that the existence of a bilingual advantage in EF has been doubted in the current literature. Criticism concerns amongst others a publication bias for positive results and methodological flaws in the literature which increase the chance of false positive results (Paap et al., 2015; Zhou and Krott, 2016). Paap et al. (2015) also criticized the insufficient control of covariates, what could distort results. Therefore, groups need to be comparable to control the impact of other potentially influential variables like SES (Hope, 2015). When comparing mono- with multilinguals, SES is an important factor, because bilingualism and SES independently influence EF and vocabulary in 8-year-olds (Calvo and Bialystok, 2014), reading (Duzy et al., 2014; Maitz et al., 2018), and spelling (Zöller et al., 2006). When investigating multilingual school children in Germany, this variable is of major importance, because multilingual children in a German setting stem more often from families with lower SES, what might hide a potential advantage. Roos and Schöler (2009) illustrated that an initial disadvantage of multilinguals in reading and spelling disappeared when SES and intelligence were controlled for. Certain characteristics of bilinguals can also influence the outcome in research on the bilingual advantage: for example, bilinguals with good language control (i.e., who rarely experienced unintended intrusion errors in a picture-naming task) showed better cognitive control than bilinguals who often unintenionally switched between languages (Festman and Münte, 2012).

### Language-Related Skills and Spelling

Relevant language-related skills, which are known to influence the development of reading and writing, comprise lexicon size, PA, STM and phonological recoding (i.e., RAN). We refer to lexicon size as the number of lexical entries in memory for a specific language, in this paper German. For multilinguals, each language has its own mental lexicon that contains on average a smaller amount of lexical entries compared to monolinguals (Bialystok, 2009). This may not be confused with a conceptual scoring of lexicon size, which captures the number of concepts in the mental lexicon regardless of language. Thus, multilinguals may have larger mental lexicons following this account (Holmström et al., 2016). PA describes the ability to perceive phonological structures, i.e., phonemes and syllables, and manipulate elements of spoken language. As it is essential to recognize which phonemes build a word, it is relevant for non-word spelling and for the analysis of word structures. STM stores language material in memory for a short period of time, which is why it is relevant in spelling tasks involving dictation (when words and sentences need to be remembered) or writing of non-words (unknown sound sequences are stored in STM). Phonological recoding is the ability to recode written symbols into sound-based representations. It is usually assessed with RAN tasks, which measure the ability to fluently and effortlessly access phonological information by requiring participants to name letters, objects or pictures under speeded conditions (Wile and Borowsky, 2004). RAN as an automatized (i.e., fast, accurate, and effortless) skill is an important precondition for fluent and efficient spelling (Meyer et al., 1998).

These four language-related skills are known predictors of reading and spelling (for reviews see Hippmann, 2008; Verhoeven et al., 2011), however their influence depends on the language and its grapheme–phoneme consistency (Moll et al., 2014). Phonological STM has been found to predict spelling accuracy in Norwegian (Lervåg and Hulme, 2010) and in English (Caravolas, 2004). Interestingly, in a comparative study of English first and second language learners, their spelling performance was influenced by different components (Jongejan et al., 2007): verbal WM was most important for first language users, whereas for second language learners it was RAN. Specifically for German primary school, Ennemoser et al. (2012) found PA and RAN to predict both spelling and reading in the first four school years.

Lexicon size plays a special role, since it predicts literacy directly (Verhoeven and Perfetti, 2011), but it also plays an important role in the development of PA and consequently influences literacy indirectly. Different theories assume that phonological representations within an initially small mental lexicon are holistic (Lonigan et al., 2013; Goodrich et al., 2014). At this point, lexicon size is an important predictor for literacy. Only with increasing lexicon size do the phonological representations become gradually more segmented and fine-grained, what is necessary to become aware of the phonological structure of words (see Metsala and Walley’s *lexical restructuring model*, 1998, and Ziegler and Goswami’s psycholinguistic *grain size theory, 2006*). This awareness allows children to use PA as resource for literacy development and the influence of lexicon size is being reduced (Metsala and Walley, 1998).

#### The Role of Lexicon Size for Multilinguals

Compared to monolinguals, multilinguals have a smaller lexicon in each language (for a review see Bialystok, 2009; for receptive skills, Bialystok and Luk, 2012). The root of this disparity does not lie in an impairment or language talent, but rather in the reduced amount of learning opportunities, for example due to a later start of German acquisition or a lower amount of exposure to each language (Pearson et al., 1997; Segerer et al., 2013; Gagarina and Klassert, 2018). Indeed, this is especially pronounced in multilingual children in Germany who have on average a lower level of language proficiency in German—the language of schooling—than their monolingual peers (Niklas et al., 2011; Klassert et al., 2014). Dissimilarities in reading and writing between mono- and multilinguals can be attributed to this difference in German lexical proficiency, as the impact of lexicon size on literacy performance is particularly strong for multilinguals. Especially their lower lexical abilities (Segerer et al., 2013; Limbird et al., 2014) influence writing both directly and indirectly, as described above.

Two types of studies verified the strong effect of lexicon size in multilinguals: first, comparisons between mono- and multilinguals showed that the latter were disadvantaged in tasks that required language-specific lexical knowledge (e.g., word spelling or reading), but not in less language-dependent measures (e.g., non-word spelling or reading; Weber et al., 2007; Segerer et al., 2013; Kormi-Nouri et al., 2015). Secondly, regression analyses showed that lexicon size was the most important predictor of bilinguals’ but not of monolinguals’ reading skills (Limbird et al., 2014).

#### The Role of PA for Multilinguals

Some researchers assume that multilinguals have greater PA skills (Campbell and Sais, 1995), because of their refined metalinguistic awareness (the ability to explicitly reflect upon language structure and meaning) built up by the experience of learning and managing two or more languages in life (Adesope et al., 2010). Another explanation might be a superior intrinsic sensitivity to language structure, due to their greater total vocabulary size (taken all languages together), improved attention to language (Bialystok and Herman, 1999) or the transferability of PA from one language to another (see, e.g., Lindsey et al., 2003). However, experimental results rarely support this assumption (for a review, see Jongejan et al., 2007). For example, Bialystok et al. (2003) found no advantage for bilinguals in a number of PA tasks, besides for phoneme segmentation for Spanish–English compared to Chinese–English bilinguals and English monolinguals. Laurent and Martinot (2010) found better PA in fourth and fifth, but not in third grade. Reasons for this discrepancy can be the participants’ age, for example because formal literacy education influences PA; also, the specific languages and language combinations influence PA, as certain linguistic features are more prominent in one language than in another. Finally, PA skills can transfer from one language to another (Jongejan et al., 2007). Due to these mixed findings the advantage of multilinguals in PA remains a contentious topic.

Studies with German samples could not find an advantage for bilinguals either (Duzy et al., 2013; Janssen et al., 2013), besides Limbird and Stanat (2006) who reported an advantage of multilinguals over monolinguals for pseudoword segmentation in German second graders, but no group differences in any other measurements of PA and at other time points. Despite the lack of differences in PA, Limbird and Stanat (2006) hypothesized in their study that the influence of PA on multilinguals’ reading should be smaller than for monolinguals. They confirmed this hypothesis and attributed it to the multilinguals’ at least numerically higher PA and on average lower reading abilities. In the study by Harrison et al. (2016), English first and second language learners were compared among others on a PA task on which they performed similarly; PA was found to be the only predictor for spelling in English first language learners, while for second language learners, PA and RAN predicted their spelling performance.

### Research Focus

The goal of the present study is to determine which variables influence spelling in mono- and multilingual primary school children. Spelling involves cognitive and linguistic factors and is influenced by general background factors, such as SES. However, to our knowledge, there are no studies contrasting the influence of EF and language on spelling between mono- and multilinguals. With respect to spelling, we measured both spelling of words (which relies mainly on the lexical route) and spelling of non-words (which is based on the non-lexical route). This allows us to compare spelling abilities when German knowledge is involved (in word spelling) and when language-specific influences are minimized (in non-word spelling) (cf. Weber et al., 2007).

For cognitive factors we considered EF, namely switching, inhibition and WM (following the seminal study by Miyake et al., 2000). Until now, scientific investigations focused only on monolinguals and showed that these EF components affected spelling performance or literacy more generally. Crucially, we extended the existing research by including bi-/multilingual children.

In the linguistic domain, we focused on two language-related skills, lexicon size in German and PA. These are influential predictors of monolinguals’ spelling (Verhoeven et al., 2011). However, these factors seem to influence multilinguals’ spelling in different ways (for English, see Jongejan et al., 2007; Harrison et al., 2016), but their role when spelling in German is unknown.

Interestingly, EF and language-related skills (can) develop differently in mono- and multilingual children. Therefore, the experience of acquiring additional languages may alter the impact of both, language and cognition, on literacy development in these groups. For multilinguals this concerns specifically on the one hand benefits in EF (see the so-called “bilingual advantage”) and PA, but on the other hand detrimental effects due to a reduced lexicon size in each language, as described above.

Our approach to investigate the role of cognitive and language skills was the following: first, we attempted to include a representative sample of today’s heterogeneous school population in Germany and investigated group performance on EF and language-related skills as well as possible differences on background variables (SES, age, intelligence). Second, we wanted to find out if the three distinct EF components exerted a unique influence on spelling in these two language groups. Due to the heterogeneity of our sample, we investigated which factors influence the groups’ performances rather than comparing the differences in strength of these relations. Third, we wanted to determine the influence of language-related skills on spelling in both groups. Therefore, we focused on lexicon size and PA, because multilingualism can influence their development. And by analyzing if the impact of EF remained when language-related skills were included in the same analyses, we finally aimed at revealing whether EF or language played the predominant role in third-grade mono- and multilinguals’ spelling performance.

#### Predictions

We predicted similar performance of both groups on tasks which were—in contrast to assessments of lexicon size and word spelling—less language-dependent (e.g., non-word spelling, intelligence, PA; see Kormi-Nouri et al., 2015). Contrarily, we expected between-group differences in German lexicon size, word spelling and SES, as a disadvantage for multilingual children has been repeatedly shown for these factors or was likely (in the case of spelling; cf. Schründer-Lenzen and Merkens, 2006). Moreover, we expected no bilingual advantage on EF task performance, because of detrimental effects especially due to SES.

Despite a lack of a bilingual advantage, we assumed the influence of cognitive skills on spelling to be different between the language groups. Drawing on the literature on monolingual children, we predicted an impact mainly of switching, but no influence of inhibition and WM (as these has been found to influence rather high-level writing processes). Switching might influence word spelling more strongly than non-word spelling, since switching between alternative spelling routes (lexical and non-lexical) might be necessary. For non-words, in contrast, only phoneme-grapheme-conversion can be applied.

Comparing the impact of language-related skills and EF, we hypothesized that EF would still play a less important role for spelling than language-related skills, which are probably more relevant at the children’s current developmental stage as writers (focusing on lower-level writing skills). The impact of language-related skills on spelling might be different between the groups. Limbird et al. (2014) found that German lexicon size was a stronger predictor for reading in multilinguals than monolinguals. Accordingly, we also expected lexicon size in German to be the strongest predictor for multilinguals’ spelling of words and to influence monolingual’ word spelling less strongly. On non-word spelling, lexicon size should not have an influence, because it requires phonemic spelling only. Concerning PA, we expected it to affect both groups and both spelling tasks similarly, extending Ennemoser et al.’s (2012) observation for monolingual primary school children.

## Materials and Methods

### Participants

Our sample consisted of 69 monolingual (33 female) and 57 multilingual children (30 female). All children attended third grade and their mean age in months was *M* = 109.1, *SD* = 7.2; see Table 1 for descriptive statistics for all tasks and background variables of both language groups. Monolingual children spoke only German at home and had no further contact with another language. Multilingual children spoke at least one other language at home (referred to as L1) besides German and had at least good verbal proficiency in their L1 (for more detail, see section “Background Variables”). The group to which we refer as *multilinguals* consisted of bilingual (*n* = 50) and trilingual students (*n* = 7). Multilinguals spoke 21 different languages as L1 with Turkish (*n* = 18) or Arabic (*n* = 8) forming the largest subgroups. Overall, 83% of the multilingual group and 7% of the monolingual group indicated a migration background (meaning at least one parent or the child was born outside of Germany). Data for this study were selected from a larger data set of 168 third graders from different schools in Germany. Children needed to be excluded from this analysis if they could not be unambiguously assigned to one language group according to their language background or had an IQ (measured with the Culture Fair Intelligence Test Scale 1, Weiß and Osterland, 2013) below 70 indicating intellectual disabilities (DIMDI, 2016).

**Table 1:** Descriptive statistics for spelling, EF, language-related skills, and other background factors per language group.

	Monolinguals		Multilinguals		
			
	*M*	(*SD*)	*M*	(*SD*)	Significance
n/females	69/48%		57/53%		
Age	107.6	(4.8)	111.0	(9.0)	^∗^
Word spelling	9.5	(3.9)	11.0	(4.2)	^∗^
Non-word spelling	4.8	(2.7)	5.3	(3.0)	
Switching	3.8	(1.2)	3.8	(1.3)	
Inhibition	1083.8	(176.5)	1032.9	(144.0)	
WM	1407.8	(275.4)	1438.2	(262.2)	
Lexicon	25.1	(6.1)	16.3	(8.4)	^∗∗∗^
PA	5.8	(2.9)	6.0	(2.7)	
STM	15.4	(3.4)	15.2	(3.5)	
RAN	2.1	(0.4)	2.0	(0.4)	
Intelligence	35.3	(5.5)	35.3	(5.0)	
SES (mother)	3.8	(1.0)	3.1	(1.5)	^∗∗^
SES (father)	3.8	(1.0)	3.2	(1.7)	^∗^
German proficiency	3.8	(0.4)	3.6	(0.6)	^∗^


*Mean scores (with standard deviations) and group differences calculated with two-tailed *t*-tests (Significance ^∗∗∗^*p* < 0.001, ^∗∗^*p* < 0.01, ^∗^*p* < 0.05) between mono- and multilinguals. [Mean values refer to: Age – in months; word spelling, non-words spelling – word errors; switching – number of experienced rules; inhibition – interference effect in ms; WM – mean reaction times in ms; lexicon, PA, STM, RAN, intelligence – number of correct answers; SES – ISCED; German proficiency – rating between 1 (*none*) and 4 (*very good*)].*

The study and the protocol were approved by the ethics committee of the University of Potsdam (approval nr. 11/2015), the head of the schools, the *Ministry of Education, Youth and Health* (Land Brandenburg) and the *Senate for Education, Youth and Science* (Berlin). The study was carried out in accordance with the ethical standards laid down in the Declaration of Helsinki. We recruited participants through class teachers who distributed written information for the parents about the study in all third-grade classes at three inclusive primary schools. The parents gave written informed consent before the beginning of the study. All participants had normal or corrected-to-normal vision, were naive as to the purpose of the study and received small gifts for their participation.

### Materials

#### Spelling

##### Word spelling

In the word spelling task, children were asked to produce the correct orthographic writing of single words. We used the respective subtest of the German standardized test battery BUEGA (Esser et al., 2008). It comprises 17 items with varying phonological and morphological complexity. Items were prerecorded and presented via loudspeaker to guarantee equal testing conditions for all participants. The number of incorrectly spelled words was used as measure of task performance.

##### Non-word spelling

To contrast children’s word spelling performance (which is confounded with their knowledge of German) with spelling purely based on phoneme–grapheme conversion, we designed a non-word spelling task. Here, children were required to produce the phonologically correct spelling of a non-word. This test comprised 12 non-words of varying length (2–3 syllables; see Supplementary Material). Quasi-universal stimuli were constructed by using (a) CV and CVC syllable structures (to avoid German-specific structures like consonant clusters), and (b) phonemes that are shared in most languages (to minimize the influence of rare or language-specific sounds such as [x] in German). The selection of phonemes, based on Maddieson (2013a,b), resulted in the following consonants: voiceless fricatives (i.e., [f], [s]), voiceless plosives (i.e., [p], [t], [k]), nasals (i.e., [m], [n]), the lateral approximant [l], and the three vowels [a], [i], and [u] that represent the three extreme points on the vowel diagram (International Phonetic Association, 2015). To guarantee equal testing conditions for all children, all non-words were prerecorded and presented via loudspeaker. The number of incorrectly spelled non-words served as a measure of non-word spelling.

#### Executive Functions

##### Switching

The Wisconsin Card Sorting Test (WCST) was chosen to assess mental set shifting in cognitive control, i.e., to infer the implied rule in card sorting guided by feedback from the environment, to change the rule when necessary and to apply the correct rule continuously (Spreen and Strauss, 1998). We used an online version of the WCST (adapted from Piper et al., 2012). The task required children to sort 64 cards according to one of three different rules (color, number, or shape) by tapping on the corresponding digital card stack on the tablet screen. As mentioned before, the current rule was not revealed but needed to be derived from visual feedback, which followed each answer. After 10 correct trials in a row, a rule change was signaled by three pink exclamation marks appearing for 750 ms. There was no time limit for answers, but children were encouraged to answer as quickly as possible. Instructions and examples were given orally to the whole group before the beginning of the training. The number of experienced rules was used in this study as a measure of switching, since the more successfully the children answered, the more rule changes they experienced.

##### Inhibition

To test participants’ ability to inhibit interfering information (i.e., color) within visually presented stimulus material, we administered the Bivalent Shape Task (BST, Mueller and Esposito, 2014). In this test, children sorted shapes (circles and squares) by pressing corresponding buttons on a tablet screen. The more salient color of the shapes defined to which condition a stimulus belonged: (a) In the neutral condition, black outlines were presented, (b) in the congruent condition, shape and color (red and blue) matched the respective answer button, and (c) in the incongruent condition, colors were interchanged, so that the shape corresponded to the correct button, but the color corresponded to the incorrect button. The incongruent condition required inhibition to refrain from pressing the incorrect button. The experiment consisted of three uniform blocks in a fixed order (neutral, followed by congruent and incongruent) comprising 20 randomized trials, and one mixed block comprising 30 randomized trials, 10 of each condition. The test began with oral instructions and examples, followed by a practice block (12 randomized trials) with visual feedback. In the experimental blocks, no feedback was given, and each item appeared immediately after button press or after a time limit of 3 s. We adapted the BST (Mueller and Esposito, 2014) to counterbalance the position (right and left) and color (blue and red) of the answer buttons over participants. As measure of inhibition, we used the interference effect (reaction time difference between incongruent and congruent trials) of the mixed blocks.

##### Working memory

To assess participants’ WM, we chose the n-back task that requires the ability to temporarily store information in memory, process it and continuously update the stored information. In this task, single letters (A, B, O, R, and S) were presented one-by-one in the center of the tablet screen. Children were asked to press a button at the bottom of the screen when the displayed letter was the same as two trials ago (two-back). Thus, they had to compare the newly perceived information with older already stored information. The task comprised two blocks with 20 pseudorandomized items of which eight were critical items (no more than two critical items in a sequence). Response times were restricted to 4 s in which the stimulus was presented for 2 s and an inter-stimulus interval with a blank screen appeared for 2 s. The task started with 10 practice trials including visual feedback, but afterward no feedback was given. The mean reaction time was used as a measure of WM.

#### Language-Related Skills

##### Lexicon size

To capture the size of the expressive lexicon in German, we used a standardized online picture naming task, the WWT (short version test 2, Glück, 2011). This test consisted of 40 colored items eliciting nouns, verbs or opposites of adjectives. It was self-paced, but time restricted, and administered on tablet computers. The number of correct answers was used as a measure of lexicon size.

##### Phonological awareness

As the participants were already in third grade, we chose a task which measured the level of PA at a higher degree of difficulty (rather than phoneme identification, segmentation, and syntheses; see Anthony and Francis, 2005). We wanted to assess complex phonological skills measured by sub-syllabic tasks and used an inversion test which relied on manipulative abilities of phonemic knowledge, namely the phoneme-inversion subtest of the standardized German test BAKO (subtest 4, Stock et al., 2003). The prerecorded items were presented one-by-one. The task required children to invert the first two sounds in 11 items (six German words and five non-words) and pronounce it out loud. The output measure was the number of correct answers.

##### Phonological short-term memory

A non-word repetition task was used to measure the children’s STM, since the ability to retain and repeat increasingly longer verbal stimuli serves as indicator of STM capacity. STM was tested with the non-word repetition task from the ZLT-II (Petermann et al., 2013). This task consists of 30 non-words built of meaningless CV-syllables and thereby avoids language-specific structures like consonant clusters. Items were prerecorded to standardize testing conditions and pronounced with a neutral prosody (equal stress on each syllable) to reduce German-specific prosodic patterns. The non-words were presented with increasing length, from two to six syllables. Children were asked to repeat each non-word after its presentation. The number of correctly repeated non-words served as a measure of STM.

##### Rapid automatized naming

Rapid automatized naming of letters was used to measure phonological recoding. More specifically, we assessed naming speed of an array of letters, which indexes the ability to effectively access and retrieve phonological entries of graphemes. RAN is considered to be a measure of automaticity (Strattman and Hodson, 2005). We chose the RAN test from the standardized German test battery TEPHOBE (Mayer, 2013). Participants received a sheet of paper with 50 letters and were required to name them in sequence as fast and as accurately as possible. The number of correct answers per second indexed naming speed of letters.

#### Background Variables

##### Questionnaires

Background information on the children and their family were derived from a paper–pencil questionnaire filled out by the parents at home (a German and a Turkish version of the questionnaire were available). To measure the family’s SES we asked for each parent’s highest school and professional qualification, which was then categorized by the ISCED (International Standard Classification of Education, UNESCO Institute for Statistics, 2012) on a scale from 0 to 6. Information on the child’s language background, the beginning of his/her acquisition of German and the languages spoken at home and in kindergarten were obtained in the questionnaire. The parents’ rating of their child’s oral language skills (i.e., mean rating for speaking and comprehension on a scale of 1 = *none* to 4 = *very good*) served as a measure of language proficiency.

##### Intelligence

To assess the participants’ non-verbal intelligence, in particular their ability to recognize and continue figurative relationships and logical sequences, we used the Culture Fair Intelligence Test Scale 1 (CFT 1-R, Weiß and Osterland, 2013). We assessed these skills with three subtests, namely Matrix, Series, and Classification. This standardized, non-verbal test was specifically chosen, because it is a culture-independent and language-free test, and consequently, it should not disadvantage children from different cultural backgrounds or with poorer German skills. The sum of correct answers served as a measure of intelligence.

### Procedure

This study is part of a larger project comprising the parents’ questionnaire and three experimental sessions. We report here only the tasks relevant for this study: in a first group session, children completed the word spelling, inhibition, and switching task on a tablet computer (Microsoft Surface Pro 2 Tablet, display size: 25.5 cm × 17 cm, resolution: 2160 px × 1440 px). In the second group session, we administered the test for non-word spelling and WM on the same tablet, and the intelligence test with a paper–pencil version. In the third, individual session, we included all tasks which required recording children’s verbal responses, i.e., German lexicon size, PA, STM, and RAN.

In group sessions, up to 14 children participated and were supervised by trained experimenters who ensured a quiet atmosphere, correct administration of tests, and understanding of instructions despite language, comprehension, or processing speed difficulties.

### Data Analysis

Data preparation for the EF tasks included exclusion of single participants whose performance was below chance or who did not participate in one of the tasks (switching: two monolinguals; inhibition: two multilinguals; WM: five multilinguals). Afterward, for the BST and N-back, reaction times of correct responses were log-transformed to normalize distributions, and outliers in form of single data points were removed by visual inspection of the reaction time distribution for each task (inhibition: single blocks with less than four correct answers and in total, 0.1% of all data points; WM: in total 0.9% of all data points). For all other tasks that included verbal responses (that concerns lexicon, PA, STM, and RAN), audio-files were transcribed. These data and the paper-pencil tests (i.e., intelligence, word and non-word spelling) were rated by one- and double-checked by another person to obtain correct ratings for all items in every tasks.

All calculations were run on R version 3.2.2 (R Core Team, 2015). In a first step, we compared all measures between the language groups with two-tailed *t*-tests to reveal performance differences in cognitive and language measures as well as in background variables. Then, we calculated correlations between the tasks measuring spelling, EF, language-related skills and the potentially influential factors intelligence and SES, for each language group separately. Spearman correlation coefficients were computed with the *rcorr* function from the *Hmisc* package (Harrell, 2017). Finally, we calculated linear mixed effects models with *glmer* function from the *lme4* package (Pinheiro et al., 2015) separately for each spelling task and for each group to find out which predictors influenced mono- and multilinguals in these tasks. We decided not to calculate one model with multilingualism as a predictor besides EF and language tasks, due to the heterogeneity of our sample and group differences (e.g., in age and migration status) that we could not control without overfitting this model. Independent variables were the raw values of the spelling tasks (correct/incorrect rating for every item) and random intercepts for participants and item were calculated to control individual differences and effects on an item-level (e.g., increasing fatigue or the impact of a preceding trial that was answered correctly or wrong; Baayen et al., 2008). As dependent variables, we used the same variables in every step for both groups and all outcome measures. These were z-transformed to allow for comparison between the predictors. First, the influence of the three EF components was tested. Secondly, those EF with significant influence were entered into the models together with the linguistic predictors. These comprised German lexicon size and PA, since multilingualism potentially influences their development or impact on spelling. In a third step, SES was added (note that we do not report these last results in detail, since model fit did not improve by adding SES. For more detail, see below). Other factors like intelligence were not entered into the models to avoid overfitting them and due to intercorrelations between cognitive, language and background factors (see below). Model fit for the generalized mixed-effect models was estimated with the marginal (*R*^2^m; variance explained by the fixed effects) and conditional coefficient (*R*^2^c; variance explained by fixed and random effects). Both were calculated with the r.*squaredGLMM* function from the package *MuMIn* version 1.41.0 (Burnham and Anderson, 2002; Nakagawa and Schielzeth, 2013).

## Results

### Group Comparisons

Group comparisons between mono- and multilinguals for all variables are displayed in Table 1. (For results of error rates in the EF tasks, see Supplementary Table 1.) As can be seen in Table 1, monolinguals and multilinguals did not differ on most variables. They performed equally on the non-word spelling test, on all three EF tasks (switching, inhibition, working memory), on most tasks testing language-related skills, i.e., PA, STM and RAN, as well as on intelligence.

Contrastingly, monolinguals produced significantly less errors in word spelling. Regarding language-related factors, they had a significantly larger expressive lexicon than their multilingual peers. Moreover, the groups differed on a number of other background variables such that monolinguals were significantly younger and showed significantly higher SES (mother’s and father’s ISCED), and parents rated their monolingual children’s proficiency in German significantly higher than did parents of multilingual children.

### Correlations

The correlation coefficients of mono- and multilinguals’ performance in spelling, cognitive, and language tasks as well as background factors are presented in Table 2. First, the results show that the correlation between the two spelling tasks (i.e., more errors in spelling words correlated with more errors in spelling non-words) was much higher for monolinguals (*r* = 0.61, *p* < 0.001) than for multilinguals (*r* = 0.38, *p* < 0.001).

**Table 2:** Correlation coefficients of monolinguals (below diagonal) and multilinguals (above diagonal) for spelling, EF, language-related skills, and other background factors.

	1	2	3	4	5	6	7	8	9	10	11
(1) Words		0.38^***^	-0.15	-0.27^*^	0.06	-0.37^***^	-0.38^***^	-0.24	-0.42^***^	-0.28^*^	-0.17
(2) Non-words	0.61^***^		-0.20	-0.10	0.02	-0.23	-0.4^***^	-0.48^***^	-0.44^***^	-0.13	-0.16
(3) Switching	-0.25^*^	-0.19		-0.02	0.27	0.31^*^	0.17	0.32^*^	-0.07	0.28^*^	0.16
(4) Inhibition	-0.07	0.17	0.02		-0.11	0.19	-0.11	0.20	0.12	0.16	-0.01
(5) WM	0.17	0.07	-0.20	-0.15		0.08	0.09	0.01	-0.02	0.07	0.18
(6) Lexicon	-0.31^*^	-0.38^***^	0.27^*^	-0.07	0.22		0.15	0.26	0.26	0.38^***^	0.57^***^
(7) PA	-0.46^***^	-0.37^***^	0.19	0.18	-0.23	0.24		0.34^*^	0.32^*^	0.19	0.24
(8) STM	-0.42^***^	-0.5^***^	0.12	-0.08	-0.01	0.41^***^	0.34^***^		0.16	-0.05	0.14
(9) RAN	-0.49^***^	-0.53^***^	0.16	0.05	-0.15	0.16	0.41^***^	0.26^*^		-0.02	0.33^*^
(10) Intelligence	-0.28^*^	-0.32^*^	0.45^***^	0.05	-0.17	0.37^***^	0.35^***^	0.12	0.24		0.29
(11) SES (mother)	0.01	0.00	0.10	0.01	0.16	0.36^***^	-0.03	0.28^*^	-0.11	0.02	


*Spearman correlation coefficients (Significance ^∗∗∗^*p* < 0.001, ^∗∗^*p* < 0.01, ^∗^*p* < 0.05).*

The three EF tasks did not correlate with each other, as they represent distinct EF-subfunctions. Regarding correlations of spelling with EF, we observed that for monolinguals switching correlated with spelling words (higher number of experienced rules correlated with fewer errors in spelling words; *r* = -0.25, *p* < 0.05), whereas for multilinguals it was inhibition which correlated with word spelling (larger interference effect correlated with fewer errors in word spelling; *r* = -0.27, *p* < 0.05). Apart from that, inhibition and WM hardly correlated with any of the other factors. Note that in both groups switching correlated with lexicon and intelligence (more experienced rules correlated with a larger lexicon and a higher intelligence score; monolinguals: *r* = 0.27, *p* < 0.05 and *r* = 0.45, *p* < 0.001; multilinguals: *r* = 0.31, *p* < 0.05 and *r* = 0.28, *p* < 0.05).

The four language-related measures (lexicon, PA, STM, and RAN) correlated with spelling of words and non-words in both groups (with the exception for multilinguals regarding correlations of lexicon with spelling non-words, *r* = -0.23, and STM with spelling words, *r* = -0.24): fewer errors in the spelling tasks correlated with a larger lexicon, and better PA, STM, and RAN. The language-related measures also correlated with one another in both groups, for example better performance in RAN with better PA (monolinguals: *r* = 0.41, *p* < 0.01, multilinguals: *r* = 0.32, *p* < 0.05), and better STM with greater lexicon size (but only for monolinguals: *r* = 0.41, *p* < 0.001).

Furthermore, we found relatively high intercorrelations of intelligence with several measures (for monolinguals a higher intelligence score correlated with fewer errors in both spelling tasks, more experienced rules in the switching task, larger lexicon, and better PA, whereas for multilinguals a higher intelligence score correlated with fewer errors when spelling words, better switching, and a larger lexicon; see Table 2). The correlation of higher SES with a larger lexicon was very high for multilinguals (*r* = 0.57, *p* < 0.001), but moderate for monolinguals (*r* = 0.36, *p* < 0.001). Furthermore, higher SES correlated with better STM for monolinguals and with better RAN for multilinguals.

### Predictors of Word Spelling

Our first regression model (see Table 3, upper part) explores the influence of the three EF components on word spelling. For the monolingual group (left column), switching (*b* = -0.36, *SE* = 0.16, *p* < 0.05) influenced word spelling significantly. Better performance on this EF task (i.e., more experienced rules in the WCST) was associated with less errors in the spelling of words. For multilinguals (right column), inhibition predicted performance in word spelling (*b* = -0.46, *SE* = 0.21, *p* < 0.05), with a larger interference effect being associated with fewer errors. The fixed effects in both models explained only a small amount of variance (*R^2^m* = 0.03 for monolinguals, *R^2^m* = 0.04 for multilinguals).

**Table 3:** Fixed effects of the linear mixed effects models predicting number of errors in word spelling.

		Monolinguals				Multilinguals			
			
		*b*	*SE*	*z*	Significance	*b*	*SE*	*z*	Significance
(1)	(Intercept)	0.14	0.35	0.40		0.66	0.41	1.60	
	Switching	-0.36	0.16	-2.19	^∗^	-0.27	0.23	-1.16	
	Inhibition	-0.08	0.16	-0.49		-0.46	0.21	-2.16	^∗^
	WM	0.13	0.16	0.81		0.23	0.23	1.01	
	*R^2^m/R^2^c*				0.03/0.5				0.04/0.58
(2)	(Intercept)	0.16	0.34	0.48		0.63	0.38	1.68	^+^
	Switching	-0.22	0.14	-1.53		0.06	0.18	0.34	
	Inhibition	-0.04	0.14	-0.27		-0.37	0.17	-2.17	^∗^
	Lexicon	-0.23	0.15	-1.52		-0.47	0.18	-2.58	^∗^
	PA	-0.59	0.15	-4.00	^∗∗∗^	-0.67	0.18	-3.77	^∗∗∗^
	*R^2^m/R^2^c*				0.09/0.5				0.12/0.55


*Regression models were calculated for mono- and multilinguals separately. In model (1) the three EF components are included as predictors, and predictors in model (2) are lexicon size, PA and the significant EF from (1). *R^2^m* represents the variance explained by the fixed effects and R^2^c by fixed and random effects. (Significance ^∗∗∗^*p* < 0.001, ^∗∗^*p* < 0.01, ^∗^*p* < 0.05, ^+^*p* < 0.1).*

The second regression model (see Table 3, lower part) includes inhibition and switching, because of their significance in the first model, and lexicon and PA. Word spelling in monolinguals was significantly predicted only by PA (i.e., the better their PA, the fewer spelling errors they made; *b* = -0.59, *SE* = 0.15, *p <* 0.001). For the multilingual group PA (*b* = -0.67, *SE* = 0.18, *p* < 0.001) and lexicon size (*b* = -0.47, *SE* = 0.18, *p* < 0.05) were significant predictors with better PA and a greater expressive lexicon predicting fewer spelling errors. Additionally, the impact of inhibition remained for multilinguals’ spelling performance (*b* = -0.37, *SE* = 0.17, *p* < 0.05). Both models explained now a larger amount of variance for mono- and multilinguals (*R^2^m* = 0.09 and *R^2^m* = 0.12, respectively).

Note that in a third model, we added the mothers’ ISCED to control for the impact of SES, but this did not improve model fit: for monolinguals, *R^2^m* declined from *R^2^m* = 0.09 to *R^2^m* = 0.07, and for multilinguals from *R^2^m* = 0.12 to *R^2^m* = 0.11. Therefore, we report only the models without SES here (see the Supplementary Table 3 for these additional models).

### Predictors of Non-word Spelling

For both language groups, we found an effect of switching on non-word spelling in our first model that is significant for monolinguals (*b* = -0.28, *SE* = 0.13, *p* < 0.05) and marginally significant for multilinguals (*b* = -0.36, *SE* = 0.20, *p* < 0.1). These effects indicate that the more categories the children could master in the WCST, the fewer spelling errors they produced (see Table 4, upper part). The explained variance of the fixed effects is equal in both groups, but very low (*R^2^m* = 0.02).

**Table 4:** Fixed effects of the linear mixed effects models predicting number of errors in non-word spelling.

		Monolinguals				Multilinguals			
			
		*b*	*SE*	*z*	Significance	*b*	*SE*	*z*	Significance
(1)	(Intercept)	-0.50	0.27	-1.87	^+^	-0.20	0.32	-0.62	
	Switching	-0.28	0.13	-2.12	^∗^	-0.36	0.20	-1.80	^+^
	Inhibition	0.15	0.13	1.17		-0.15	0.19	-0.81	
	WM	-0.02	0.13	-0.12		0.12	0.20	0.63	
	*R^2^m/R^2^c*				0.02/0.36				0.02/0.26
(2)	(Intercept)	-0.48	0.26	-1.86	^+^	-0.35	0.31	-1.15	
	Switching	-0.13	0.12	-1.06		-0.18	0.18	-1.06	
	Lexicon	-0.36	0.12	-2.92	^∗∗^	-0.28	0.18	-1.61	
	PA	-0.27	0.12	-2.24	^∗^	-0.49	0.17	-2.90	^∗∗^
	*R^2^m/R^2^c*				0.06/0.36				0.08/0.46
(3)	(Intercept)	-0.49	0.26	-1.91	^+^	-0.33	0.30	-1.11	
	Switching	-0.15	0.11	-1.36		-0.09	0.17	-0.56	
	Lexicon	-0.19	0.13	-1.54		-0.22	0.17	-1.34	
	PA	-0.16	0.12	-1.37		-0.36	0.16	-2.16	^∗^
	STM	-0.38	0.12	-3.05	^∗∗^	-0.47	0.18	-2.63	^∗∗^
	*R^2^m/R^2^c*				0.08/0.36				0.1/0.46


*Regression models were calculated for mono- and multilinguals separately. In model (1) the three EF components are included as predictors, in model (2) predictors are lexicon size, PA and the (marginally) significant EF from (1), and in (3) STM was added to model (2). *R^2^m* represents the variance explained by the fixed effects and R^2^c by fixed and random effects. (Significance ^∗∗∗^*p* < 0.001, ^∗∗^*p* < 0.01, ^∗^*p* < 0.05, ^+^*p* < 0.1).*

In the second model (see Table 4, in the middle), only switching was included, since WM and inhibition did not influence non-word spelling in either group in the first model. For monolinguals, we found lexicon size (*b* = -0.36, *SE* = 0.12, *p* < 0.01) and PA (*b* = -0.27, *SE* = 0.12, *p* < 0.05) to significantly predict non-word spelling performance such that the larger the lexicon and the better the PA abilities, the lower the error rate when spelling non-words. For the multilingual group, the only significant predictor of non-word spelling was PA (*b* = -0.49, *SE* = 0.17, *p <* 0.01). The explained variance of the fixed effects improved for both groups (*R^2^m* = 0.06 for monolinguals and *R^2^m* = 0.08 for multilinguals).

As for word spelling, adding the mothers’ ISCED to the regression models reduced model fit (for monolinguals: to *R^2^m* = 0.05, and for multilinguals: to *R^2^m* = 0.07). Hence, we report only the models without SES (but these additional models can be found in Supplementary Table 4).

Since the second models explained relatively little variance, we decided to run another model including STM (see Table 4, lower part). STM is responsible for short-term storage of verbal information and therefore essential to successfully perform on our non-word spelling task as children had to memorize non-words of increasing length. When STM was entered into the model, it was the strongest predictor for monolinguals (*b* = -0.38, *SE* = 0.12, *p* < 0.01) and multilinguals (*b* = -0.47, *SE* = 0.18, *p* < 0.01) revealing that the better the STM abilities, the fewer errors were made when spelling non-words. For multilinguals, additionally PA influenced non-word spelling (*b* = -0.36, *SE* = 0.16, *p* < 0.05). In sum, STM is the best predictor for non-word spelling. That neither lexicon nor PA maintained their significance for the monolinguals’ performance is partially caused by the intercorrelations between STM and lexicon (*r* = 0.41, *p* < 0.001), and STM and PA (monolinguals: *r* = 0.34, *p* < 0.001). The models with STM fit the data best, since they explained the largest amount of variance (for monolinguals *R^2^m* = 0.08 and for multilinguals *R^2^m* = 0.1).

## Discussion

We investigated the influence of EF and language-related skills on spelling in a group of mono- and multilingual third graders. By including a word and a non-word spelling task, we were able to contrast spelling based on lexical knowledge (word spelling) and phonemic spelling (non-word spelling via the non-lexical route). We assessed naturally heterogeneous groups of mono- and multilingual third graders with inherent differences in SES and lexicon size to the detriment of multilinguals. Despite these differences, the groups did not differ in their performance on non-word spelling, PA and EF, whereas monolinguals outperformed multilinguals in the word spelling and German lexicon test. Our regression analyses revealed that switching explained a small amount of variance in word and non-word spelling for monolinguals. Contrastingly for multilinguals, inhibition influenced word spelling and a trend indicated an influence of switching in non-word spelling. When we added lexicon size and PA—two important predictors of literacy—to our models, the influence of switching disappeared in both groups and tasks, but inhibition remained as predictor for multilinguals’ word spelling. Overall, language was a better predictor for spelling and both language groups shared the most influential factors in each spelling task: PA for spelling words and STM for spelling non-words. As predicted, some language-related skills influenced only multilinguals: lexicon size predicted their word spelling, and PA influenced their non-word spelling performance.

### The Role of EF in Spelling

Since our sample consisted of very heterogeneous groups of children including multilinguals with disadvantages in SES and lexicon size, we neither expected nor found a bilingual advantage in any of the three EF components. From the literature on the bilingual advantage we know that differences in EF between language groups tend to appear only when groups are well-matched to reduce the influence of confounding variables (Hope, 2015). After all, many variables influence EF (Diamond, 2013), and bi- or multilingualism is only one of them. As Morton and Harper (2007) showed, an apparent bilingual advantage could stem from a hidden advantage in SES from which bilinguals’ EF benefited. In our study, the opposite was the case, that is, multilinguals’ disadvantage in SES most likely balanced out the possible positive effects of multilingualism, since both influenced EF (Calvo and Bialystok, 2014).

In the word spelling task, we found a differential role of EF on mono- versus multilinguals. Monolinguals’ word spelling performance was influenced by switching, as we predicted, and multilinguals’ performance by inhibition. However, the impact of EF on monolinguals disappeared, when language-related skills were controlled for in our models. This can be explained by the role of the lexicon size. In contrast to multilinguals, monolinguals likely have already built up a large number of orthographic entries in their orthographic lexicon, probably linked to their better proficiency in German in general, and the larger lexicon in particular. The large orthographic lexicon enabled them to rely on their refined lexical skills for efficient processing during the spelling tasks, what freed mental resources and EF could come into play. We conclude that in primary school language skills initially play the dominant role for spelling. We further speculate that the major impact of language skills on spelling lasts until the developing language processing skills reach a certain threshold of proficiency (these might relate to the size of the lexicon, the efficiency and automaticity of processing, the well-established use of the lexical route). After this threshold has been passed, freed cognitive resources could be redirected to EF to improve coordination of parallel processes and develop toward higher-level processes of writing for which the influence of EF has been documented in the literature.

For multilinguals, the explanation of the impact of EF on spelling that we found is more difficult. We did not expect this effect of inhibition, since earlier studies (Altemeier et al., 2008; Lubin et al., 2016) with monolinguals did not find an effect of inhibition on spelling. The discrepancy between our finding and the literature might be caused by different tasks involving different processes apart from inhibition: Altemeier et al. (2008) used a Color-Word interference test, including word reading, and Lubin et al. (2016) tested inhibition with an Opposite World task, requiring children to name digits. The BST that we administered in our study is language-independent, because it involves shape identification and inhibition of color (as the more salient feature). Another possibility for our different finding is that we tested multilinguals. Additionally, the direction of the effect is somewhat counterintuitive, since a larger interference effect—indicating poorer inhibition skills—predicted less spelling errors. This relation even remained significant when language skills were statistically controlled for. The fact that we found this effect only in word spelling indicates that it is connected to processing German, the language of schooling, and not to general writing processes. To clarify this relation and replicate this finding, however, further studies are necessary.

In non-word spelling, both groups rely on the same mechanisms, because German language knowledge is not relevant for task performance: we found a significant effect of switching for monolinguals and the trend in the same direction for multilinguals. The similarity between the groups is likely caused by the minimized need for German specific processing in this task allowing multilinguals to exploit their potential regarding the use of EF. As we mentioned above, monolinguals might be able to used more EF due to their advanced German skills. Multilinguals, however, need more cognitive resources for language processing (like phoneme analysis that is necessary in non-word spelling) resulting in less influence of EF. Here, the marginally significant effect might be caused by individual differences in this group, because some advanced spellers might already have the capacity to utilize switching for spelling. It may be used as an additional resource to make spelling more efficient, since basic language processing skills proceed more automatically than in less proficient spellers.

Why is switching relevant for both spelling tasks? The influence of switching on spelling seems to be replicable for spelling in different languages. This concerns the studies by Lubin et al. (2016) for French 9-year-olds, by von Suchodoletz et al. (2017) for German pupils, and the longitudinal study by Altemeier et al. (2008) showing that rapid automatized switching predicted spelling in English in the first 3 years of school. Spellers need to switch between the ongoing parallel processes during spelling, like language processing, accessing lexical entries or phoneme–grapheme correspondences, graphomotor and output control (Lubin et al., 2016). These demands are universal for word and non-word spelling, which is why switching is the EF component relevant for word and non-word spelling.

Our finding that WM did not influence spelling is in accordance with the literature. WM likely comes into play at a later age and during more complex writing tasks, since WM influences higher-level tasks in writing, like text reviewing process that take place in advanced writers who have managed low-level writing processes (Swanson and Berninger, 1996). The spelling of single words and non-words does not require children to master these higher-level processes for successful task performance.

### Similar Main Components for Mono- and Multilinguals’ Spelling

Despite some differences in EF involvement, mono- and multilinguals shared the main components during spelling: first, PA was the most influential factor in spelling performance of words (see Table 3) and non-words (if STM is not considered, see second model in Table 4). PA is essential for phonemic writing (for non-words and unknown real words) and spelling via the lexical-route (Pfost, 2015). The ability to recognize syllables and phonemes is necessary to identify phonemes for phoneme–grapheme conversion and to determine morphemes. With regard to spelling words, our results add to the evidence provided by Ennemoser et al. (2012) who found that monolingual German primary school children relied mostly on PA for spelling in first to fourth grade (for Norwegian, see Lervåg and Hulme, 2010; for English, Caravolas, 2004) and extents this to multilinguals in German primary school.

The second main component both language groups shared was STM as strongest predictor for non-word spelling (see third model in Table 4). Non-word spelling necessarily relies on STM, because phonological information must be stored in memory during mental processing (Martin and Gupta, 2004; Repovš and Baddeley, 2006) and during non-word dictation. The relation between STM and spelling is well-documented in the literature: for example, Swanson and Berninger (1996) have determined the influence of STM on spelling of letters and words in a sample of 10- to 12-year-olds, or Wimmer and Mayringer (2002) found children with a spelling deficit to have a smaller STM capacity. Initially, the relation between STM and spelling was not in the focus of our study, since STM is not a component of EF (Miyake et al., 2000). But the poor model fit with only EF and language-related skills as predictors (see Table 4 middle part) made it necessary to investigate further influential variables.

### The Impact of Language Proficiency on Multilinguals’ Spelling

Our results confirm the greater role of lexicon size for multilinguals’ literacy. Limbird et al. (2014) found this link between lexicon size and reading in the first 3 years of primary school and we expand this association to word spelling of third graders. According to the *lexical restructuring model*, lexicon size influences spelling to a certain threshold after which it does not play a role anymore (Metsala and Walley, 1998). Only when words are stored as phonologically detailed representations, the role of lexicon size in word spelling decreases, as we could see in our monolingual group. However, many children in our multilingual group seem to be below that threshold due to their smaller lexicon size in German, meaning that they have not yet developed phonologically fine-grained lexical representations for German, their language of schooling (also found by Niklas et al., 2011; Segerer et al., 2013; Limbird et al., 2014).

Another indication of the strong impact of language-related skills on multilinguals is that language-dependent tests posed a greater challenge for multilinguals in our study. In comparison to their monolingual peers, multilinguals performed more poorly in word spelling and lexicon, but similarly on all other tasks (i.e., non-word spelling, EF, PA, STM, RAN, and intelligence) that relied as little as possible on German language knowledge. Our results are in line with other studies that found similar result patterns: Kormi-Nouri et al. (2015) found Iranian bilinguals in grade one to five to show poorer word reading, but similar non-word reading performance compared to their monolingual peers. Studies with German school children also revealed that multilinguals underperformed in language-dependent tasks but performed similarly in tests that relied less on German knowledge (Weber et al., 2007; Duzy et al., 2013; Segerer et al., 2013). To avoid an undesired impact of lexicon in testing situations with multilinguals (Messer, 2010; Parra et al., 2011), tests should involve single letters, non-words or visual items. One concrete example is our non-word spelling task, which was designed to minimize language-specific influences. In contrast to other tests using pseudowords (e.g., Hasselhorn et al., 2012), the items were based on quasi-universal structures, with common vowels and consonants, a simple syllable structure (without language-specific structures like consonant clusters) and without morphology (see task description in section “Materials”). We consider this approach essential to assess actual phoneme-based spelling performance in groups with diverse language experience and proficiency (Schöppe et al., 2013).

Interestingly, PA played a greater role in non-word spelling for multilinguals than for monolinguals. For word reading, Limbird and Stanat (2006) found the opposite pattern, as described above. PA exerts a greater impact on multilinguals in our study, because they likely have less fine-grained phonological representations due to their smaller lexicon size (Metsala and Walley, 1998). Non-word spelling relies entirely on the non-lexical route which requires correct phoneme identification to translate phonemes into graphemes. Consequently, multilinguals with more holistic lexical representations might have problems identifying phonemes correctly, what makes PA a more important predictor for their non-word spelling performance compared to monolinguals.

### Limitations

Studying the interplay between further potentially important factors on spelling is still necessary; this concerns for example SES, migration background and the lower language status of migrant languages in Germany (Plewnia and Rothe, 2011). In our study, the impact of SES on multilinguals’ language skills is supported by the higher correlation between lexicon and SES for multilinguals than monolinguals, but SES did not add to explaining spelling performance beyond EF and language skills. However, multilinguals’ disadvantage in SES has negative repercussions on language skills (Calvo and Bialystok, 2014), literacy (Roos and Schöler, 2009) and school success in general (Zöller et al., 2006), because parents with higher SES are more likely to provide early literacy activities, a stimulating educational input, like access to media, experiences and multiple and diverse language learning opportunities. The latter are especially important for multilingual children who need to acquire German often outside their home (Zöller et al., 2006).

Our choice of EF tasks relied on the tripartite model of EF (Miyake et al., 2000), but we need to acknowledge that the strict division of EF in three separate components has been questioned in the literature (Friedman and Miyake, 2017). Moreover, the precise measurement of EF components with one task has been criticized due to *task impurity* (Friedman, 2016). Task impurity stems from superficial factors like stimuli characteristics (e.g., words versus pictures) or response modality (e.g., motoric versus verbal) that might alter characteristics of an EF task. This concerns especially the WCST as measure of switching, because it is a quite complex task (Best and Miller, 2010): it comprises three stimulus categories, three possible rules, and it requires problem solving strategies to reveal the new rules, inhibition of inappropriate responses and of irrelevant stimuli characteristics, etc. (Miyake et al., 2000; Diamond, 2013). It is therefore possibly a more general measure of EF, but further investigations should verify the role of switching in spelling with other experimental designs.

Our analyses do not allow us to compare the strength our predictors have on spelling in mono- versus multilinguals. For example, Limbird et al. (2014) found PA to influence reading more strongly in monolinguals than multilinguals, but we cannot draw this kind of conclusions from our data with regard to spelling. Therefore, more comparable groups need to be investigated and the differential influence of for example EF needs to be calculated in one model. We refrained from this strategy, because of the heterogeneity of our language groups concerning the differences in migrations status and age that could not be controlled for without risking to overfit regression models.

## Conclusion

We studied a naturally heterogeneous sample of mono- and multilingual third graders in Germany, with multilinguals having on average a lower SES and smaller German lexicon size. In our study, we contrasted the influence of cognitive (i.e., the EF components switching, inhibition and WM) and language factors (i.e., lexicon size and PA) on word and non-word spelling in these groups. EF explained only a small amount of variance in both spelling tasks in both groups. Switching predicted monolinguals’ word spelling, whereas word spelling in multilinguals was predicted by inhibition. In non-word spelling, both groups shared switching as the only predictor (the impact for multilinguals was only marginally significant). Since the effect of switching disappeared when language was controlled for, we postulate that language processing initally takes up more cognitive resources that are not available for EF. This is the case for multilinguals for whom language factors play a predominant role in spelling due to their smaller German lexicon size. Beyond this threshold, language processing (e.g., lexical access, phoneme–grapheme conversion) is so fluent that cognitive resources are freed and EF become more influential—the monolinguals in our study are likely at this developmental stage.

Comparing the impact of language and cognition, we found that language-related skills exerted a greater influence on spelling than EF and mono- and multilinguals shared the main predictors for spelling: PA in word spelling and STM in non-word spelling. Our study also replicated the strong role of lexicon size for multilinguals’ word spelling. This relation needs to be considered when comparing mono- with multilinguals, since many tests use verbal stimuli or depend on language in other ways, what potentially disadvantages multilinguals.

## Author Contributions

SC, AK, and JF contributed equally to the design and preparation of the study as well as to data acquisition. SC carried out the statistical analysis, and SC and JF composed the article. All authors read and approved the final manuscript. This study was carried out in collaboration between the authors.

## Conflict of Interest Statement

The authors declare that the research was conducted in the absence of any commercial or financial relationships that could be construed as a potential conflict of interest.
