# Supplementary material for: Executive Functions and Language: Their Differential Influence on Mono- vs. Multilingual Spelling in Primary School
Source: Front Psychol. 2019 Feb 6;10:97. doi: 10.3389/fpsyg.2019.00097 (PMC6374619; doi:10.3389/fpsyg.2019.00097)
Supplement: Supplementary file 1 [file Data_Sheet_1.docx]

Supplementary materials

Table 1. *Error rates in percentage for the EF tasks*

|  |  | Monolinguals | | Multilinguals | |  |
| --- | --- | --- | --- | --- | --- | --- |
|  |  | *M* | *(SD)* | *M* | *(SD)* | *p* |
| Switching | total errors | 22 | (12) | 24 | (12) | .37 |
| Inhibition | uniform neutral | 2 | (4) | 2 | (3) | .71 |
|  | uniform cong. | 3 | (4) | 3 | (4) | .63 |
|  | uniform incong. | 9 | (9) | 7 | (7) | .29 |
|  | mixed neutral | 5 | (9) | 5 | (8) | .95 |
|  | mixed cong. | 4 | (8) | 3 | (7) | .37 |
|  | mixed incong. | 14 | (15) | 11 | (11) | .24 |
| WM | 1-back com. | 3 | (3) | 4 | (6) | .17 |
|  | 1-back om. | 8 | (5) | 7 | (5) | .92 |
|  | 2-back com. | 7 | (7) | 7 | (5) | .73 |
|  | 2-back om. | 18 | (7) | 19 | (6) | .41 |

Note. Mean scores (with standard deviations) and group differences calculated with two-tailed t-tests between mono- and multilinguals. (cong. - congruent, incong. - incongruent, com. - commissions, om. - omissions)

Table 2. *Stimulus items for the nonword spelling task, with increasing complexity.*

|  | Nonword | N syllables | N phonemes |
| --- | --- | --- | --- |
| 1. | li-fu | 2 | 4 |
| 2. | nal-mi | 2 | 5 |
| 3. | kut-mip | 2 | 6 |
| 4. | tis-naf | 2 | 6 |
| 5. | ki-ma-lu | 3 | 6 |
| 6. | ka-mi-fu | 3 | 6 |
| 7. | pus-ka-mi | 3 | 7 |
| 8. | saf-li-pun | 3 | 8 |
| 9. | lus-mip-ta | 3 | 8 |
| 10. | nal-tuf-mik | 3 | 9 |
| 11. | tis-kun-pam | 3 | 9 |
| 12. | suk-lap-nif | 3 | 9 |

Note. Nonwords consisting of CV-syllables (e.g. li, fu) and CVC-syllables (e.g. nal, kut).

Table 3. *Fixed effects of the linear mixed effects model predicting number of errors in word spelling.*

|  | Monolinguals | | | | Multilinguals | | | |
| --- | --- | --- | --- | --- | --- | --- | --- | --- |
|  | *b* | *SE* | *z* | Significance | *b* | *SE* | *z* | Significance |
| (Intercept) | 0.10 | 0.34 | 0.29 |  | 0.65 | 0.40 | 1.64 |  |
| Switching | -0.14 | 0.15 | -0.94 |  | 0.16 | 0.20 | 0.77 |  |
| Inhibition | -0.06 | 0.14 | -0.44 |  | -0.26 | 0.19 | -1.36 |  |
| Lexicon | -0.13 | 0.16 | -0.81 |  | -0.43 | 0.24 | -1.75 | ^+^ |
| PA | -0.61 | 0.17 | -3.58 | *** | -0.71 | 0.20 | -3.62 | *** |
| SES | 0.00 | 0.16 | 0.01 |  | -0.11 | 0.23 | -0.46 |  |
| *R^2^m/R^2^c* |  |  |  | .07/.48 |  |  |  | .11/.56 |

Note. Regression models were calculated for mono- and multilinguals separately. (*** *p* < .001, ** *p* < .01, * *p* < .05, ^+^ p < .1)

Table 4. *Fixed effects of the linear mixed effects model predicting number of errors in non-word spelling.*

|  | Monolinguals | | | | Multilinguals | | | |
| --- | --- | --- | --- | --- | --- | --- | --- | --- |
|  | *b* | *SE* | *z* | Significance | *b* | *SE* | *z* | Significance |
| (Intercept) | -0.47 | 0.26 | -1.81 | ^+^ | -0.34 | 0.33 | -1.02 |  |
| Switching | -0.08 | 0.12 | -0.63 |  | -0.20 | 0.20 | -0.96 |  |
| Lexicon | -0.33 | 0.13 | -2.50 | * | -0.25 | 0.25 | -1.01 |  |
| PA | -0.30 | 0.13 | -2.26 | * | -0.46 | 0.19 | -2.47 | * |
| SES | 0.07 | 0.12 | 0.56 |  | -0.06 | 0.22 | -0.26 |  |
| *R^2^m/R^2^c* |  |  |  | .05/.35 |  |  |  | .07/.48 |

Note. Regression models were calculated for mono- and multilinguals separately. (*** *p* < .001, ** *p* < .01, * *p* < .05, ^+^ p < .1)
